# The Effect of Fine (PM_2.5_) and Coarse (PM_10-2.5_) Particulate Matter on Survival Outcomes in Nasopharyngeal Carcinoma Patients: A Retrospective Cohort Study in a Northern Thailand Tertiary Care Center

**DOI:** 10.3390/jcm15197367

**Published:** 2026-09-22

**Authors:** Weerachai Watcharatsiriyuth, Phakhinee Leesirisopha, Pichit Sittitrai, Hanpon Klibngern, Tapanut Ariyanon, Orapan Sangiamporn, Donyarat Ruenmarkkaew

**Affiliations:** Department of Otolaryngology, Chiang Mai University, Chiang Mai 50200, Thailand; waiweerachai@gmail.com (W.W.); titas.337@gmail.com (P.L.); psittitrai@yahoo.com (P.S.); hanponk@gmail.com (H.K.); tapanut.a@cmu.ac.th (T.A.); orapan.sa@cmu.ac.th (O.S.)

**Keywords:** fine particulate matter, coarse particulate matter, PM_2.5_, nasopharyngeal carcinoma, survival

## Abstract

**Objective:** This study aimed to examine the association between exposure to ambient fine (PM_2.5_) and coarse (PM_10-2.5_) particulate matter and survival outcomes among patients with nasopharyngeal carcinoma. **Methods:** A retrospective study was conducted among patients with nasopharyngeal carcinoma. Annual mean levels of ambient particulate matter and gaseous air pollutants were assessed from the Thailand Pollution Control Department. The Cox proportional hazards model was used to estimate the association between exposure to PM_2.5_ and PM_10-2.5_, categorized into four concentration quartiles, and survival outcomes. **Results:** One hundred and forty patients were included. Five-year overall survival (OS) and disease-free survival (DFS) were 73.1% and 61.9%, respectively. Univariable and multivariable analyses showed that increased PM_2.5_ exposure was not significantly associated with OS or DFS. In multivariable analysis, higher PM_10-2.5_ exposure categories were associated with poorer survival outcomes. Compared with patients in the lowest exposure quartile, those in the highest PM_10-2.5_ exposure quartile demonstrated significantly poorer OS (aHR 8.12, 95% CI 1.30–15.27) and DFS (aHR 9.17, 95% CI 4.58–13.98) when comparing the 4th with the 1st quartiles of concentration. **Conclusions:** High exposure to PM_10-2.5_ was associated with an increased risk of death and disease recurrence in patients with nasopharyngeal carcinoma, with statistically significant associations observed between the highest and lowest exposure quartiles.

## 1. Introduction

Nasopharyngeal carcinoma (NPC) is a malignant tumor arising from the epithelial cells of the nasopharynx and is relatively rare worldwide. However, its incidence is markedly higher in certain geographic regions, particularly in Southern China and Southeast Asia, including Thailand [1,2]. The etiology of NPC is multifactorial, involving complex interactions among genetic susceptibility, Epstein–Barr virus infection, and environmental exposure [2].

Ambient air pollution, particularly fine particulate matter with a diameter less than 2.5 μm (PM_2.5_), has been extensively studied for its adverse health effects. Long-term exposure to PM_2.5_ has been associated with respiratory and cardiovascular diseases and carcinogenic processes, with strong evidence of an association with lung cancer [3]. With regard to upper aerodigestive tract malignancies, a study using the SEER database reported associations between PM_2.5_ and an increased incidence of head and neck cancers [4]. For NPC specifically, available evidence suggests potential adverse oncologic effects associated with air pollution exposure. A cohort study from Southern China reported an increased risk of disease recurrence associated with long-term exposure to PM_2.5_ and its chemical components [5]. A population-based study in Taiwan found that exposure to high concentrations of PM_2.5_ and nitrogen dioxide was associated with a higher incidence of NPC [6]. More recent studies from China have also reported associations between exposure to multiple air pollutants, including PM_2.5_, black carbon, and ozone, and increased mortality among patients with NPC, particularly at higher exposure levels [7,8]. While PM_2.5_ can deposit in the lower airway and alveoli, coarse particulate matter with a diameter between 10 and 2.5 μm (PM_10-2.5_) tends to deposit in the upper aerodigestive tract, including the nasopharynx [9,10,11], making NPC a biologically plausible target for this type of exposure [9,12]. Although evidence linking PM_10-2.5_ to NPC is weaker than the established relationship between PM_2.5_ and lung cancer, a Taiwanese population-based study reported a significant association between long-term PM_10-2.5_ exposure and increased NPC risk after adjustment for PM_2.5_ and major confounders [9]. In addition, increasing evidence suggests that ambient particulate matter not only contributes to carcinogenesis but may also adversely influence tumor progression, mortality, and survival following cancer diagnosis, particularly in lung cancer and NPC [5,8,13]. Proposed mechanisms include chronic inflammation, oxidative stress, DNA damage, epigenetic dysregulation, immune modulation, and alterations in the tumor microenvironment [12,14]. However, most mechanistic evidence supporting these pathways comes from lung cancer studies, experimental models, and respiratory disease research rather than directly from NPC [5,8,13]. Therefore, the biological interpretation of PM_10-2.5_-related survival effects in NPC remains largely extrapolative, although the preferential deposition of PM_10-2.5_ within the nasopharyngeal mucosa provides biological plausibility for such effects. Furthermore, studies evaluating PM_10_- or PM_10-2.5_-related survival outcomes remain limited across many cancer sites and are particularly scarce for NPC [15,16].

NPC is the most common head and neck cancer in Thailand [17]. In Northern Thailand, open biomass burning, including agricultural residue burning and forest fires, during the dry season, particularly from January to April, appears to have played a major role in air quality over the past decade [18]. These sources of pollutant emissions have been the main contributors to ambient particulate matter (both PM_2.5_ and PM_10-2.5_) and to toxic gaseous pollutants, such as sulfur dioxide (SO_2_), nitrogen dioxide (NO_2_), and ozone (O_3_) [18,19]. Although the aforementioned studies suggest that exposure to PM_2.5_ adversely affects NPC development and mortality, data on survival outcomes remain limited, and, in particular, the effect of PM_10-2.5_ has not been studied. Therefore, the aim of this study was to examine the association between exposure to ambient PM_2.5_ and PM_10-2.5_ and survival outcomes among patients with NPC treated at our institute, the largest tertiary care center in Northern Thailand.

## 2. Materials and Methods

### 2.1. Study Design

This was a retrospective study of patients aged 18–90 years who were diagnosed with nasopharyngeal carcinoma and treated at our center between 1 January 2019 and 31 December 2022. The medical records were accessed from hospital archives. Data pertinent to patients were excluded if they had concurrent or previous malignancy or had metastatic disease at initial diagnosis.

All procedures contributing to this work complied with the ethical standards of the relevant national and institutional guidelines on human experimentation and with the Declaration of Helsinki 1975, revised in 2013. The study protocol was approved by the Ethics Committee of the Faculty of Medicine, Chiang Mai University (reference number 273/2025). As patient identities were anonymized, the Ethics Committee waived the requirement for informed consent.

### 2.2. Clinical Variable Assessment

Demographic and clinical data were collected, including age at diagnosis, gender, smoking status, alcohol consumption, ECOG performance status, comorbidities, tumor stage according to the American Joint Committee on Cancer staging system (8th Edition), and treatment modality. Treatment modalities were categorized into three groups by primary treatment strategy: radiotherapy alone, concurrent chemoradiotherapy, and sequential chemoradiotherapy. The latter is defined as concurrent chemoradiotherapy followed by adjuvant chemotherapy or induction chemotherapy followed by concurrent chemoradiotherapy.

### 2.3. Air Pollution Assessment

Mean annual levels of ambient particulate matter (PM_2.5_ and PM_10_) and toxic gaseous air pollutants, including sulfur dioxide (SO_2_), nitrogen dioxide (NO_2_), and ozone (O_3_), were assessed using data obtained from the Thailand Pollution Control Department. Concentrations were assigned based on district-level monitoring data corresponding to the recorded residential address of each patient. Average annual particulate matter and gaseous pollutant concentration exposure of each patient from the diagnostic year through the last follow-up year were calculated. Concentrations of PM_10-2.5_ were calculated by subtracting the measured concentrations of PM_2.5_ from those of PM_10_ [9].

### 2.4. Outcome Assessment

Overall survival (OS) was defined as the time from the start of treatment to death from any cause. Disease-free survival (DFS) was defined as the time from the start of treatment to the first documented disease recurrence or death from any cause. Patients for whom no event was recorded were censored on 31 December 2025 or at the date of last follow-up, whichever occurred first.

### 2.5. Statistical Analysis

Baseline characteristics were summarized using descriptive statistics. Continuous variables are presented as mean ± standard deviation or median (interquartile range), depending on the data distribution. Because different concentrations of air pollutants may have different impacts on survival outcomes, the annual average concentration of each pollutant was categorized into four quartile-based groups, defined as the 1st, 2nd, 3rd, and 4th concentration groups.

Overall survival and disease-free survival were estimated using the Kaplan–Meier method. A univariable Cox proportional hazards model was used to estimate hazard ratios (HRs) and 95% confidence intervals (CIs) for the association between exposure to PM_2.5_ and PM_10-2.5_, stratified by four concentration quartiles, and survival outcomes. Then, a multivariable Cox regression model was constructed, adjusting for predefined clinically relevant variables and gaseous pollutants. A *p*-value of <0.05 was considered statistically significant for all statistical analyses. The analyses were performed using IBM SPSS Statistics for Windows (version 20.0, IBM Corp., Armonk, NY, USA).

## 3. Results

### 3.1. Patient Characteristics

One hundred and forty patients were included in the analysis. The mean age was 53.7 ± 13.3 years; there was a higher frequency of males, with a ratio of 2.2:1. Most patients (75.7%) were in the advanced stage (stage III–IV), and the majority of all patients (83.6%) were treated with sequential chemoradiotherapy. Demographics and clinical characteristics of the whole cohort are presented in Table 1.

### 3.2. Environmental Pollutants

Median concentrations of PM_2.5_ and PM_10-2.5_ from the year of diagnosis to the year of last follow-up of each patient were 30.74 (IQR, 24.24, 33.22) μg/m^3^ and 14.54 (IQR, 10.53, 18.93) μg/m^3^, respectively. Details of environmental pollutant concentrations are shown in Table 2.

### 3.3. Survival Outcomes

The median follow-up duration was 52 months (range, 8–84 months). During follow-up, 34 deaths and 51 disease-free survival events (disease recurrence and/or death) were observed. Across the whole cohort, 3-year and 5-year OS were 87.3% and 73.1%, while 3-year and 5-year DFS were 71.8% and 61.9%, respectively (Figure 1).

### 3.4. Correlation Between Survival Outcomes and PM_2.5_ and PM_10-2.5_ Exposure

Univariable Cox proportional hazards model analysis was performed to evaluate the association between quartile-based PM_2.5_ and PM_10-2.5_ exposure categories and survival outcomes. The results demonstrated that PM_2.5_ exposure was not significantly associated with OS or DFS. In contrast, higher PM_10-2.5_ exposure quartiles were associated with poorer DFS. Specifically, patients in the 4th concentration group were significantly associated with lower DFS than those in the 1st concentration group (HR 3.73, 95% CI 1.58–8.78, *p* = 0.003) (Table 3).

In the multivariable analysis, adjustments were made for clinical covariates, including age (<50 vs. ≥50 years), gender (female vs. male), ECOG performance status (0–1 vs. 2), smoking status (never vs. ever), alcohol consumption (never vs. ever), underlying diseases (diabetes mellitus, hypertension, and chronic obstructive pulmonary disease), AJCC stage (I–II vs. III–IV), treatment modality (RT vs. CCRT vs. sequential CRT), and gaseous pollutant covariates, including quartile stratification of SO_2_, NO_2_, and O_3_. PM_2.5_ exposure was not significantly associated with OS or DFS. However, patients in higher PM_10-2.5_ exposure quartiles were associated with poorer OS and DFS. Compared with the 1st concentration group, patients in the 4th concentration group were significantly associated with poorer OS (aHR 8.12, 95% CI 1.30–15.27, *p* = 0.031). In addition, compared with the 1st concentration group, patients in the 3rd and 4th concentration groups were significantly associated with poorer DFS (aHR 6.00, 95% CI 1.01–11.92, *p* = 0.043 and aHR 9.17, 95% CI 4.58–13.98, *p* = 0.001, respectively) (Table 3).

## 4. Discussion

Five-year overall and disease-free survival for the whole cohort were 73.1% and 61.9%, respectively, which were consistent with previous reports on NPC survival (62–84% and 61.6–78.8%, respectively) [17,20]. This study demonstrated associations between higher PM_10-2.5_ exposure quartiles and poorer OS and DFS. These associations were particularly significant between the highest and lowest groups after adjusting for relevant clinical and gaseous pollutant covariates. One possible explanation for the observed association is that PM_10-2.5_ tends to deposit within the nasopharynx [9,10,11], where it may contribute to local inflammation, oxidative stress, and DNA damage, biological pathways that have been implicated in carcinogenesis and tumor progression [3,21], as demonstrated in previous studies [9]. In addition, PM_10-2.5_ has been hypothesized to alter the tumor microenvironment and may be associated with biological processes linked to tumor invasiveness, metastatic potential, and adverse survival outcomes [3,21,22].

Most previous studies reported an association between high PM_2.5_ exposure and increases in NPC incidence and mortality [5,6,7,8]. In contrast, in this study, PM_2.5_ exposure quartiles were not significantly associated with either OS or DFS in univariable or multivariable Cox proportional hazards analyses. In addition, a study from Taiwan found that NPC incidence was significantly associated with exposure to PM_10-2.5_ but not to PM_2.5_ [9]. Notably, the median PM_2.5_ concentration in this study was comparable with those of previous studies (30.74 μg/m^3^ versus 32.71–33 μg/m^3^, respectively) [7,8,9].

Regarding gaseous pollutants, a previous study reported an association between O3 and NPC mortality [7]. The current study found that only SO_2_ showed a decreasing DFS trend, with an HR of 2.85 (95% CI, 1.18–6.87; *p* = 0.020), comparing the 4th with the 1st quartile groups. However, after adjustment for potential clinical and pollutant covariates, multivariable Cox proportional hazards model analysis showed that the association was attenuated and no longer statistically significant (Table 3).

In addition to conventional clinicopathological factors, metabolic tumor characteristics derived from [18F]FDG PET/CT have emerged as important prognostic biomarkers in NPC. Recent evidence from Quartuccio et al. demonstrated that higher baseline SUVmax, metabolic tumor volume (MTV), and total lesion glycolysis (TLG) were associated with poorer response to neoadjuvant chemotherapy in patients with locally advanced nasopharyngeal carcinoma [23]. These findings suggest that tumor metabolic activity and burden may provide prognostic and predictive information beyond traditional staging systems. Because PET/CT-derived metabolic parameters were not consistently available in our retrospective cohort, they were not included in the current analysis. Future studies incorporating both environmental exposure measures and PET/CT-derived biomarkers may help clarify the interplay between air pollution, tumor biology, treatment response, and survival outcomes.

This study has several limitations that should be considered when interpreting the findings. First, the retrospective single-center design may be subject to residual confounding and selection bias. Although multivariable analyses were performed to adjust for clinically relevant variables and gaseous pollutants, several important prognostic factors, including plasma Epstein–Barr virus (EBV) DNA levels, EBV status, and other detailed tumor burden characteristics, were not consistently available and therefore could not be included in the analyses. Second, the relatively small sample size and limited number of outcome events may have reduced statistical power and increased the risk of model overfitting, particularly given the number of clinical and environmental covariates included in the multivariable Cox models. During follow-up, 34 overall survival events and 51 disease-free survival events were observed. Given the relatively large number of estimated model parameters, the resulting events-per-variable ratio was modest and may have contributed to coefficient instability, inflated effect estimates, and wider confidence intervals. This limitation may partially explain the relatively large adjusted hazard ratios observed for some PM_10-2.5_ exposure categories. Consequently, these estimates should be interpreted cautiously and considered exploratory rather than definitive. Third, exposure assessment was based on district-level ambient air pollution monitoring data linked to patients’ residential addresses rather than individual-level exposure measurements. Residential history during follow-up was unavailable; therefore, patients were assumed to remain at the same residence throughout the study period. Information regarding residential relocation, occupational exposures, commuting patterns, household air pollution, and time spent indoors versus outdoors was also unavailable. Consequently, actual individual exposure may have differed from the estimated district-level ambient exposure, potentially resulting in non-differential exposure misclassification. In addition, average pollutant concentrations were calculated from the year of diagnosis through the last follow-up year, resulting in exposure assessment periods that varied according to each patient’s survival time. Consequently, the exposure metric was partially determined by follow-up duration and may have been related to the outcome process itself. This approach introduces the potential for time-dependent exposure bias, whereby differences in survival duration could influence the exposure estimates used in the analyses. Therefore, the observed associations should be interpreted cautiously and should not be considered evidence of a causal effect of post-diagnosis particulate matter exposure. Future prospective studies using predefined exposure windows and longitudinal time-varying exposure assessments are needed to address this limitation. Finally, because multiple statistical comparisons were performed across several pollutants, exposure quartiles, and survival outcomes, the possibility of type I error cannot be excluded. Accordingly, these findings should be considered exploratory and hypothesis-generating and require validation in larger multicenter prospective studies with longer follow-up periods, more refined exposure assessment, and incorporation of additional prognostic biomarkers.

## 5. Conclusions

The results suggest that higher PM_10-2.5_ exposure was associated with poorer overall survival and disease-free survival among patients with nasopharyngeal carcinoma, with statistically significant associations observed between the highest and lowest exposure quartiles. Given the retrospective design, limited number of outcome events, potential model instability, possible time-dependent exposure bias, and exposure misclassification, these findings should be interpreted cautiously and should not be considered evidence of causality. Rather, they should be regarded as exploratory observations that warrant validation in larger prospective multicenter studies.

## Figures and Tables

**Figure 1:**
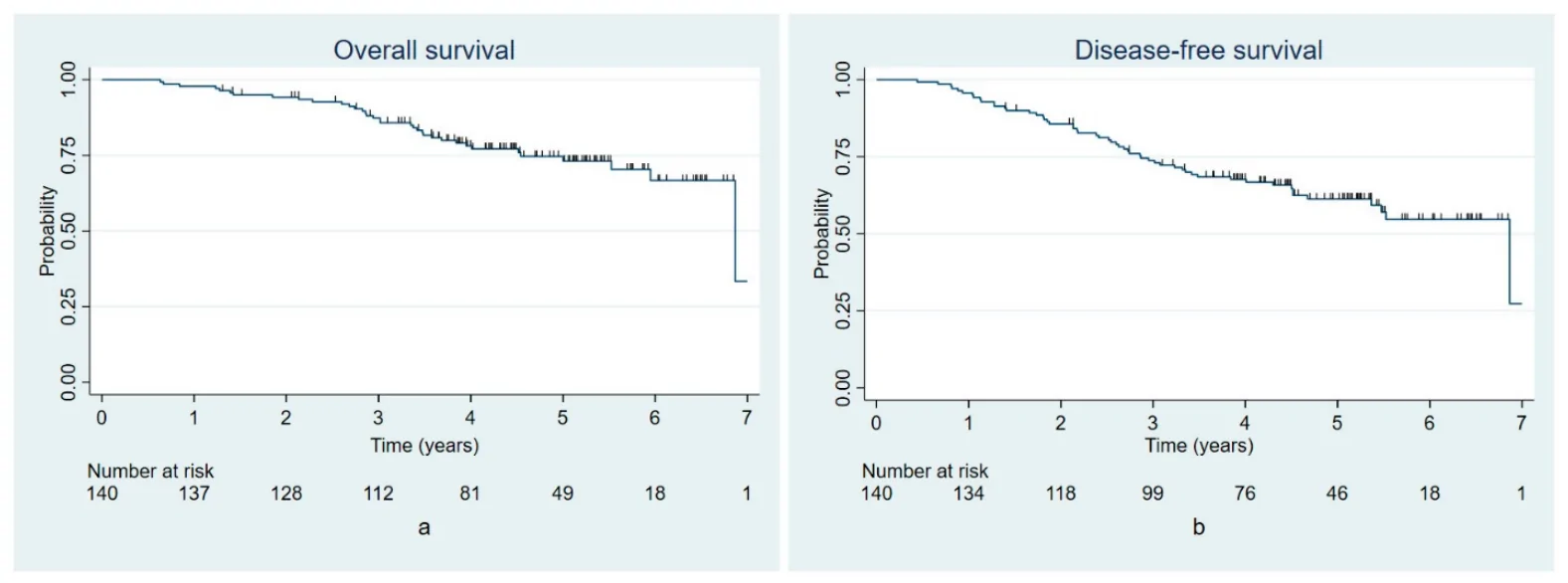
Kaplan–Meier plot for overall survival (**a**) and disease-free survival (**b**) of the entire cohort.

**Table 1:** Demographic, clinicopathologic, and treatment characteristics of the study participants.

Characteristics	Number (*n* = 140)	%
Age (years)		
<50	57	40.7
≥50	83	59.3
Gender		
Female	44	31.4
Male	96	68.6
ECOG performance status		
0–1	135	96.4
2	5	3.6
Smoking		
Never	85	60.7
Ever	55	39.3
Alcohol consumption		
Never	72	51.4
Ever	68	48.6
Diabetes mellitus		
No	128	91.4
Yes	12	8.6
Hypertension		
No	115	82.1
Yes	25	17.9
Chronic obstructive pulmonary disease		
No	137	97.9
Yes	3	2.1
WHO classification of carcinoma		
NKSCC	139	99.3
KSCC	1	0.7
AJCC staging		
I–II	34	24.3
III–IV	106	75.7
Treatment modality		
RT	9	6.4
CCRT	14	10
Sequential CRT	117	83.6

ECOG, The Eastern Cooperative Oncology Group; NKSCC, non-keratinizing squamous cell carcinoma; KSCC, keratinizing squamous cell carcinoma; RT, radiotherapy; CCRT, concurrent chemoradiotherapy; CRT, chemoradiotherapy.

**Table 2:** Environmental pollutant concentration exposure of the entire cohort stratified by quartile.

Air Pollutants	1st Quartile Group	2nd Quartile Group	3rd Quartile Group	4th Quartile Group
PM_2.5_ (μg/m^3^)	<24.24	2.24 to <30.74	30.74 to <33.22	≥33.22
PM_10-2.5_ (μg/m^3^)	<10.53	10.53 to <14.54	14.54 to <18.93	≥18.93
PM10 (μg/m^3^)	<37.61	37.61 to <46.26	46.26 to <52.09	≥52.39
SO_2_ (ppb)	<1.07	1.07 to <1.40	1.40 to <1.89	≥1.89
NO_2_ (ppb)	<4.43	4.43 to <6.88	6.88 to <9.01	≥9.01
O_3_ (ppb)	<18.82	18.82 to <24.39	24.39 to <26.64	≥26.64

ppb, parts per billion.

**Table 3:** Univariable and multivariable (clinical and pollution covariate adjustment) Cox proportional hazards model analyses of PM_2.5_ and PM_10-2.5_ stratified by quartile concentration predicting overall survival and disease-free survival.

Variables	Overall Survival				Disease-Free Survival			
	HR (95% CI)	*p*-Value	aHR (95% CI)	*p*-Value	HR (95% CI)	*p*-Value	aHR (95% CI)	*p*-Value
PM_2.5_								
1st quartile group	1 (reference)		1 (reference)		1 (reference)		1 (reference)	
2nd quartile group	0.43 (0.17–1.08)	0.072	0.25 (0.06–1.02)	0.113	0.81 (0.36–1.84)	0.616	0.77 (0.24–2.49)	0.663
3rd quartile group	0.44 (0.18–1.07)	0.071	0.39 (0.09–1.46)	0.157	0.61 (0.25–1.45)	0.259	0.79 (0.21–2.95)	0.722
4th quartile group	1.15 (0.14–1.89)	0.427	0.92 (0.02–2.63)	0.513	1.19 (0.57–2.49)	0.645	0.83 (0.20–3.40)	0.796
PM_10-2.5_								
1st quartile group	1 (reference)		1 (reference)		1 (reference)		1 (reference)	
2nd quartile group	1.03 (0.36–2.94)	0.959	1.52 (0.25–9.18)	0.650	1.15 (0.42–3.19)	0.784	1.67 (0.33–8.51)	0.537
3rd quartile group	1.27 (0.46–3.49)	0.649	7.75 (0.69–86.39)	0.096	2.31 (0.93–5.72)	0.071	6.00 (1.01–11.92)	0.043
4th quartile group	2.10 (0.84–5.28)	0.144	8.12 (1.30–15.27)	0.031	3.73 (1.58–8.78)	0.003	9.17 (4.58–13.98)	0.001
SO_2_								
1st quartile group	1 (reference)		1 (reference)		1 (reference)		1 (reference)	
2nd quartile group	1.14 (0.44–2.96)	0.786	1.19 (0.34–4.25)	0.780	1.29 (0.48–3.46)	0.619	1.54 (0.47–5.09)	0.477
3rd quartile group	0.82 (0.29–2.28)	0.706	0.27 (0.06–1.29)	0.101	2.26 (0.94–5.46)	0.070	1.06 (0.29–3.85)	0.925
4th quartile group	1.69 (0.69–4.18)	0.253	0.33 (0.06–1.94)	0.219	2.85 (1.18–6.87)	0.020	0.28 (0.06–1.32)	0.108
NO_2_								
1st quartile group	1 (reference)		1 (reference)		1 (reference)		1 (reference)	
2nd quartile group	0.29 (0.09–1.19)	0.334	0.16 (0.03–1.89)	0.136	0.44 (0.16–1.20)	0.109	0.14 (0.30–1.69)	0.215
3rd quartile group	0.61 (0.24–1.53)	0.292	0.25 (0.04–1.61)	0.144	1.28 (0.58–2.79)	0.543	0.24 (0.04–1.33)	0.101
4th quartile group	0.99 (0.44–2.26)	0.989	0.37 (0.53–2.59)	0.318	1.65 (0.78–3.50)	0.189	0.25 (0.04–1.42)	0.116
O_3_								
1st quartile group	1 (reference)		1 (reference)		1 (reference)		1 (reference)	
2nd quartile group	1.03 (0.39–2.69)	0.953	1.75 (0.46–6.64)	0.412	0.66 (0.29–1.49)	0.324	1.22 (0.43–3.47)	0.703
3rd quartile group	0.94 (0.35–2.53)	0.904	0.94 (0.25–3.57)	0.924	0.92 (0.43–1.96)	0.831	0.91 (0.36–2.29)	0.844
4th quartile group	1.67 (0.67–4.17)	0.275	1.98 (0.54–7.24)	0.300	1.07 (0.50–2.29)	0.858	0.78 (0.29–2.09)	0.616

HR, hazard ratio; 95% CI, 95% confidence interval; aHR, adjusted hazard ratio.

## Data Availability

The data sets generated during and/or analyzed during the current study are available from the corresponding authors on reasonable request.
